# Development and validation of a high-resolution T2WI-based radiomic signature for the diagnosis of lymph node status within the mesorectum in rectal cancer

**DOI:** 10.3389/fonc.2022.945559

**Published:** 2022-09-16

**Authors:** Gesheng Song, Panpan Li, Rui Wu, Yuping Jia, Yu Hong, Rong He, Jinye Li, Ran Zhang, Aiyin Li

**Affiliations:** ^1^ Department of Radiology, The First Affiliated Hospital of Shandong First Medical University, Jinan, China; ^2^ Department of Radiology, Central Hospital Affiliated to Shandong First Medical University, Jinan, China; ^3^ Department of Radiology, Shandong University, Jinan, China; ^4^ Department of Radiology, The Shandong First Medical University, Jinan, China; ^5^ Department of Radiology, Shandong Provincial ENT Hospital, Cheeloo College of Medicine, Shandong University, Jinan, China; ^6^ Marketing, Medical Technology Co., Ltd., Beijing, China

**Keywords:** magnetic resonance imaging, lymph node, radiomic signature, diagnostic imaging, rectal cancer

## Abstract

**Purpose:**

The aim of this study was to explore the feasibility of a high-resolution T2-weighted imaging (HR-T2WI)-based radiomics prediction model for diagnosing metastatic lymph nodes (LNs) within the mesorectum in rectal cancer.

**Method:**

A total of 604 LNs (306 metastatic and 298 non-metastatic) from 166 patients were obtained. All patients underwent HR-T2WI examination and total mesorectal excision (TME) surgery. Four kinds of segmentation methods were used to select region of interest (ROI), including method 1 along the border of LNs; method 2 along the expanded border of LNs with an additional 2–3 mm; method 3 covering the border of LNs only; and method 4, a circle region only within LNs. A total of 1,409 features were extracted for each method. Variance threshold method, Select K Best, and Lasso algorithm were used to reduce the dimension. All LNs were divided into training and test sets. Fivefold cross-validation was used to build the logistic model, which was evaluated by the receiver operating characteristic (ROC) with four indicators, including area under the curve (AUC), accuracy (ACC), sensitivity (SE), and specificity (SP). Three radiologists with different working experience in diagnosing rectal diseases assessed LN metastasis respectively. The diagnostic efficiencies with each of four segmentation methods and three radiologists were compared to each other.

**Results:**

For the test set, the AUCs of four segmentation methods were 0.820, 0.799, 0.764, and 0.741; the ACCs were 0.725, 0.704, 0.709, and 0.670; the SEs were 0.756, 0.634, 0.700, and 0.589; and the SPs were 0.696, 0.772, 0.717, and 0.750, respectively. There was no statistically significant difference in AUC between the four methods (*p* > 0.05). Method 1 had the highest values of AUC, ACC, and SE. For three radiologists, the overall diagnostic efficiency was moderate. The corresponding AUCs were 0.604, 0.634, and 0.671; the ACCs were 0.601, 0.632, and 0.667; the SEs were 0.366, 0.552, and 0.392; and the SPs were 0.842, 0.715, and 0.950, respectively.

**Conclusions:**

The proposed HR-T2WI-based radiomic signature exhibited a robust performance on predicting mesorectal LN status and could potentially be used for clinicians in order to determine the status of metastatic LNs in rectal cancer patients.

## Introduction

Colorectal cancer is a common digestive tract tumor, accounting for the third most common tumor in the world (1, 2). The diagnosis and treatment of rectal cancer have been improved in recent years, but the postoperative mortality remains high due to the high recurrence and metastasis rate (3–6). Lymph node (LN) metastasis is one of the most important metastatic pathways of rectal cancer (7–9). The mesorectal LN located within the mesorectal fascia is the first and most frequently involved as it is the nearest regional LN to the tumor lesion. Clarifying the LN metastasis within the mesenteric fascia can (1) help to accurately stage N and further optimize the treatment plan (10); (2) help to evaluate the circumferential margin and predict the prognosis of TME surgery (11); and (3) accurately judge the risk stratification and evaluate the recurrence risk and postoperative survival rate (10). At the same time, studies have shown that the 5-year survival rate of N0 patients is 60%–80%, while the 5-year survival rate of N + patients is only about 30% (12, 13). Therefore, it is of great clinical significance to clarify the metastasis of mesenteric LN in patients with rectal cancer (10).

**Figure 1:**
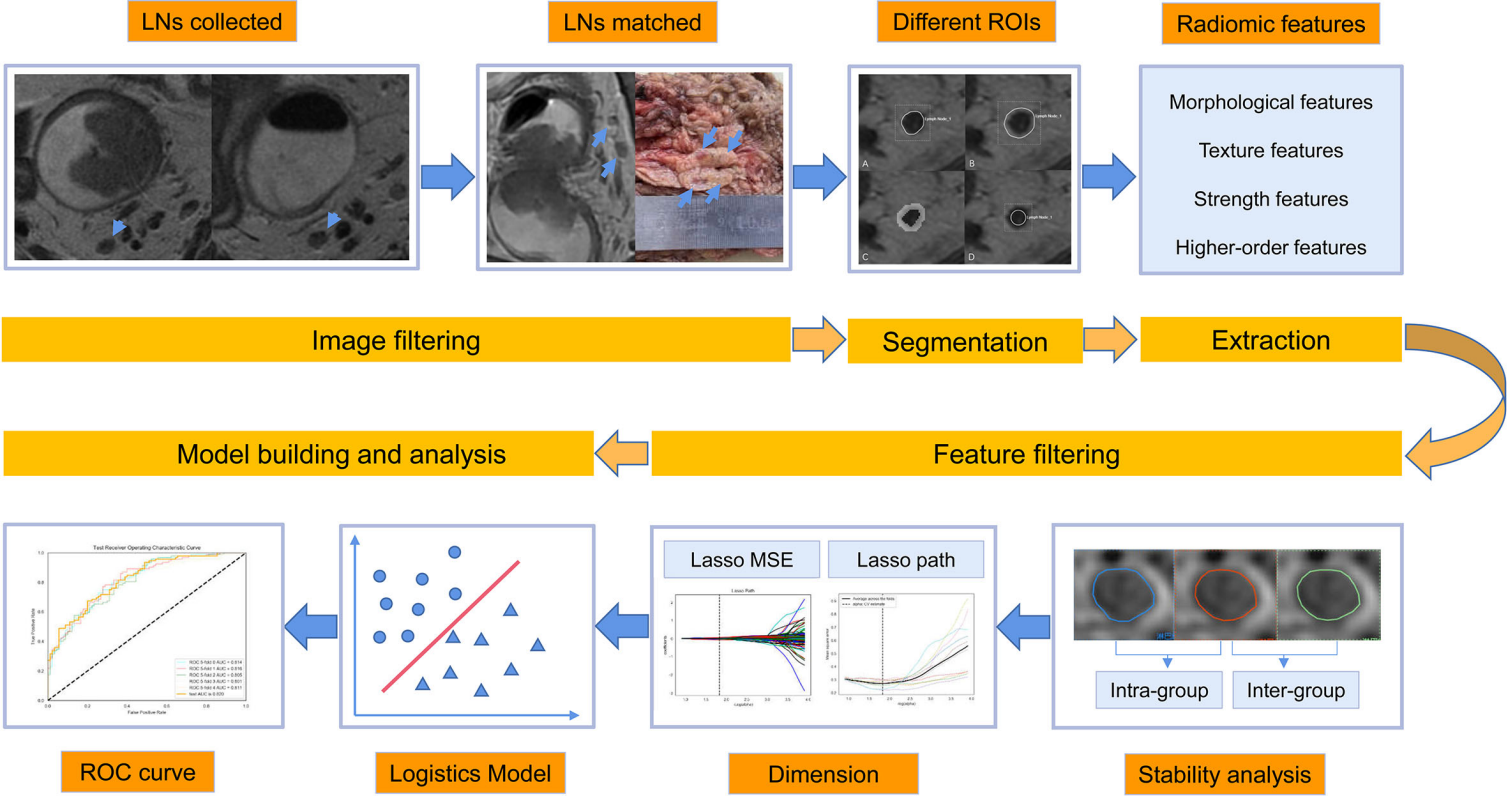
The process of imaging radiomics.

High-resolution MR (HR-MR) is an imaging examination method commonly used in clinic to evaluate the LN metastasis of rectal cancer, which can well display the main features for judging the LN metastasis including nodal size, border contour, and internal signal. However, previous studies have shown that the diagnostic efficacy of MRI in judging the involvement of LN in rectal cancer is not satisfactory (11–14). Although the size of malignant LNs increases due to the infiltration of tumor cell, the diagnostic threshold of the size remains uncertain (15–18). The diagnostic efficiency is thus not high (19–21) for the size overlap of the benign and malignant LNs. In addition to the size, the border contour and the internal signals of LNs also have diagnostic value (21–23). The morphology of the malignant LNs tend to be abnormal, such as lobulated, spiculated, and indistinct (21). At the same time, tumor tissue infiltration and necrosis or extracellular mucin is mixed with residual normal LN tissue, resulting in the inhomogeneous signal of malignant LNs (21). However, the diagnostic criteria based on border contour or internal signal are highly subjective and lack objective quantitative standards, which results in a poor consistency among observers and reduces the repeatability of diagnostic results (24). Clinically, an objective, stable, quantitative, and simple diagnostic method is needed to diagnose the status of mesorectal LNs in rectal cancer. Further improving the utilization of image information is desired to improve the diagnostic accuracy. Radiomics aims to extract a large amount of data from images, find rules through data mining to reflect the changes or differences between diseases, and achieve repeatability by screening a large number of features. Radiomics can overcome the limitations of subjective assessment of radiologists from imaging. In recent years, radiomics has been used to evaluate the T-stage, LN status, gene expression, and biological characteristics of rectal cancer preoperatively (25–29). Image segmentation is one of the most critical steps during radiomics. The best ROI segmentation method should include the most characteristic part of the lesion area (30) and should have good stability simultaneously. Therefore, in this study, radiomics based on HR-T2WI with different region of interest (ROI) segmentation methods were used to build prediction models in diagnosing the mesorectal LNs of rectal cancer. We compared the stability and the impact on the final results of different segmentation methods. Subjective diagnosis by imaging physicians was also included in the study and compared with the radiomics model. The aim of this study was to find an objective, quantitative, and simple diagnostic method for benign and malignant mesorectal LNs to improve the diagnostic accuracy. **Figure 1** shows the process of imaging radiomics.

## Materials and methods

### Establishment of patient cohort

The study was reviewed by the ethics committee of Qianfo Mountain Hospital in Shandong Province. All examinations were agreed upon by the patients with a signed informed consent form. Patients with rectal mass who came to Shandong Qianfo Mountain Hospital from June 2016 to April 2021 were collected. Exclusion criteria of MR examination (exclusion criteria 1) were as follows: having received radiotherapy or chemotherapy, being unable to tolerate MR examination (such as fever, metal substances in the body, mental history, or claustrophobia), and non-rectal cancer lesions such as adenoma, stromal tumor, or neuroendocrine tumor confirmed by preoperative colonoscopy.

### Magnetic resonance imaging acquisition

The MR scanner used in this study was GE 750 3.0 T (GE Medical Systems, Boston, USA) equipped with an abdominal coil (eight channels). The coil center was positioned at the level of the anterior superior iliac spine. The patient was in supine position with feet first. The scanning sequence and parameters of this study are listed in **Table 1**. HR-T2WI sequence was performed perpendicular to the diseased bowel. The upper part of the scanning range was more than 5 cm away from the upper edge of the tumor and the lower part was more than 2 cm away from the lower edge of the tumor, covering the resection range of TME surgery.

**Table 1:** The scanning sequences with parameters applied in this study.

sequence	TE	TR	FOV	Slice	Spacing	matrix
	(ms)	(ms)	(cm)	(mm)	(mm)	
Sagittal T2WI	85	8,137	27	4	0.4	352×352
Oblique axial HR-T2WI	102	Auto	18	3	0	320×288
CoronalT2WI	90	7832	32	4	0.4	480×480
Axial T1WI	Min Full	Auto	32	3	0.3	320×288

### Preparation before examination

Bowl preparation before MRI examination could reduce the artifacts caused by intestinal contents, intestinal peristalsis, and respiratory movement, and thus improve the image quality. The corresponding preparation procedures mainly included the following: (1) Cleaning enema to empty feces in rectum. The intrauterine device (IUD) was taken out before examination. (2) Intramuscular injection of anisodamine to reduce the motion artifacts caused by intestinal smooth muscle peristalsis. (3) Training patients to keep breathing stable to reduce the interference of abdominal motion artifacts caused by respiratory movement.

### Image screening and LN localization

After MRI examination, a radiologist (Dr. Song) with 5 years of experience in the diagnosis of rectal diseases evaluated the images. According to the MR images and the comprehensive situation of patients, the exclusion criteria were further set (exclusion criteria 2): poor image quality or serious artifacts; no LNs (short diameter, less than 3 mm) were found in the mesentery on HR-T2WI sequence; planning to undergo local resection such as Transanal Endoscopic Microsurgery (TEM); no operation conditions or receiving neoadjuvant treatment or no TME operation for other reasons.

**Figure 2:**
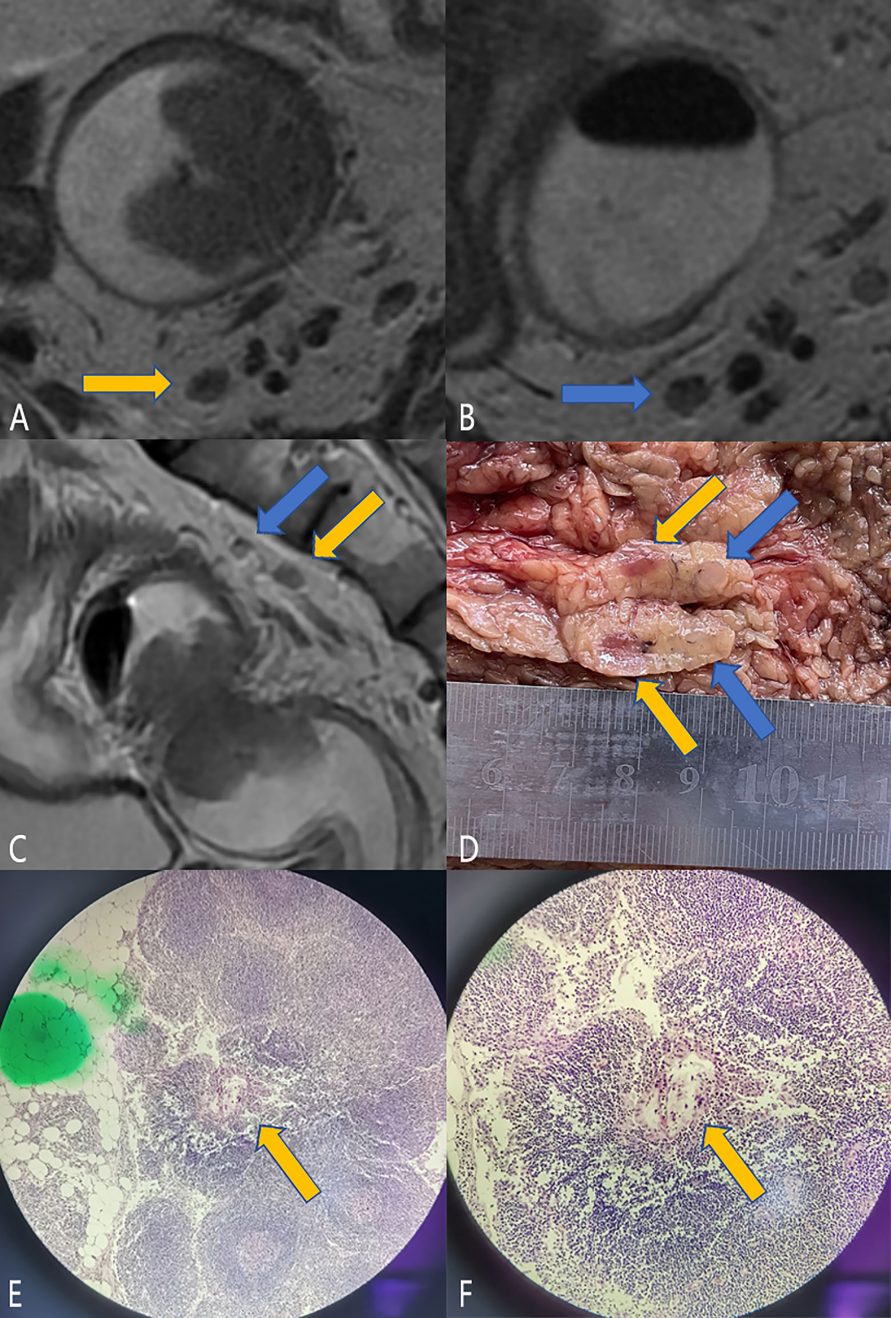
LN imaging–pathological localization process. Male, 59 years old, rectal cancer (t3n1, a regional metastatic LN, the lesion length on MR is 4.2 cm, and the pathological lesion size is 4 * 4 cm). **(A–C)** are the magnetic resonance images. Panels A and B show the two LNs (blue and orange arrows) in the upper mesentery in the oblique cross-sectional position, and panel **(C)** shows the positional relationship of the two LNs in the sagittal position (blue and orange arrows). It can be seen that the two LNs are adjacent up and down. Panel **(D)** shows the two LNs located on the resected specimen. For better display, the two LNs have been cut open. After section staining, metastasis was found in the LNs indicated by orange arrows, as shown in panels **(E, F)**. Then, the LN was successfully located and turned out to be a malignant LN. Combined with the final pathology, there was only one LN metastasis, and the remaining LNs were considered as benign LNs.

Patients underwent TME surgery within 1 week of MRI examination. After the operation, the radiologist (Dr. Song) and pathologists jointly processed the resected specimens to locate the LNs according to the relative position between the LNs, the position relationship with the intestinal wall, and adjacent small vessels. The corresponding LNs on MR and pathological specimens were included in the final study cohort. **Supplementary Figure 1** shows the establishment process of the LN cohort. **Figure 2** shows the process of LN imaging–pathological localization.

### Radiomics analysis

#### Image processing and ROI segmentation

**Figure 3:**
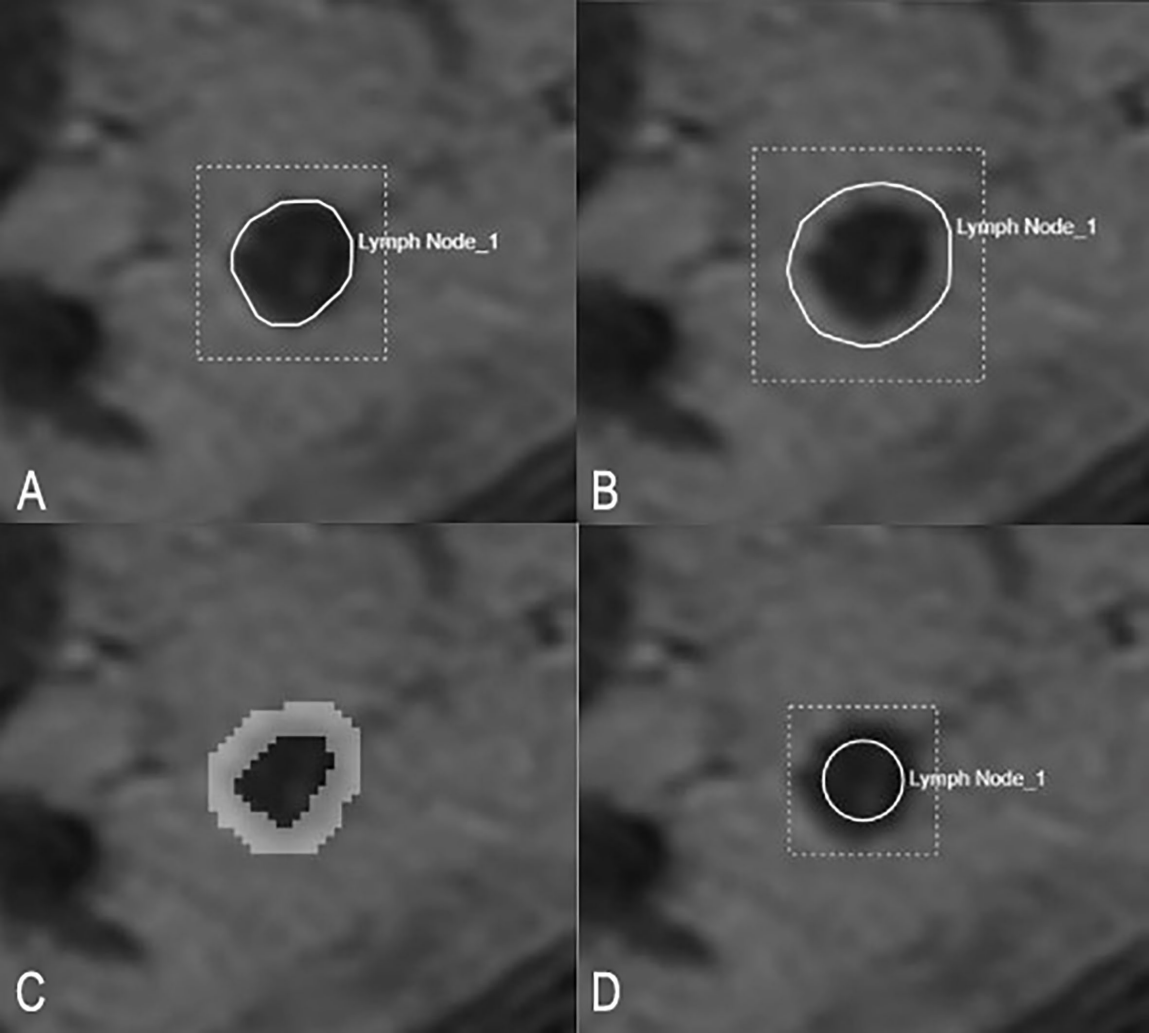
Schematic diagram of four segmentation methods. **(A)** shows the ROI along the border of LN. **(B)** shows the ROI along the border of LNs and expand outward by 2–3 mm. **(C)** shows the ROI only covering the edge of LNs. **(D)** shows the ROI as a circle located inside an LN.

The HR-T2WI of the enrolled cases in DICOM were anonymously uploaded to the HuiyiHuiying radcloud workstation [Huiying Medical Technology (Beijing) Co., Ltd.], and the short diameter of each enrolled LNs was recorded on the HR-T2WI. Four different segmentation methods were used: (1) along the contour of LNs; (2) expand about 2–3 mm along the contour of LNs; (3) annular, including only the edge of LNs; (4) round, located in the contour of LNs. **Figure 3** shows the schematic diagram of four segmentation methods.

#### Feature extraction

After segmentation, four kinds of imaging features were extracted, including morphological features, texture features, intensity features, and high-order features. Morphological features contain 14 features that describe the size of ROI, such as volume, elongation, and maximum diameter. Intensity features, texture features, and high-order features reflect the characteristics of the change law of signal or image intensity. There are 18 intensity characteristics that describe the distribution characteristics of voxel intensity in ROI, including mean, median, minimum, maximum, kurtosis, and skewness. Texture features are calculated from gray-level co-occurrence texture matrix, gray-level run length matrix (glrlm), gray-level region size matrix (glszm), gray-level dependence matrix (GLDM), neighborhood gray-level difference matrix (ngtdm), and other algorithms. In this study, there are 75 texture features, including GLCM (24 eigenvalues), GLDM (14 eigenvalues), glrlm (16 eigenvalues), and glszm (16 eigenvalues). High-order features can be obtained after filtering or wavelet transformation of intensity and texture features, in order to reduce noise and enhance image features. Many other types of filters can also be used, such as logarithm, exponential, and gradient. Finally, 1,409 eigenvalues were obtained (**Supplementary Table 1**).

#### Stability test

The correlation coefficient was used to test the consistency of features of inter- and intra-observers. Forty-five HR-T2WI of LNs were randomly selected for the test. To assess the reliability of inter-observers, a radiologist (Dr. Song) segmented the ROIs, and after 1 week, the same radiologist segmented the ROIs for the second time. To assess the reliability of intra-observers, ROI segmentation was performed independently by another radiologist (Dr. Li) and compared with the first segmentation results of the first radiologist (Dr. Song). An ICC value greater than 0.75 indicates good consistency and is included in the follow-up study. The first radiologist completes the ROI segmentation of the remaining images (31, 32).

#### Data dimensionality reduction

The dimensionality reduction of features can reduce the number of variables and maintain the useful ones with diagnostic information. Through dimensionality reduction, the noise can be removed and the computational complexity can be reduced, which makes the data set easier to use and the results easier to understand. In this study, variance threshold method, Select K Best, and Lasso algorithm were used to reduce the dimension.

#### Data grouping and model establishment

In this study, all 604 LNs were randomly divided into a training set and a test set according to the ratio of 7:3 (training set = 422, test set = 182). The ratio of benign and malignant LNs in the training set and test set was both basically 1:1 (215 malignant and 207 benign LNs in the training set, 93 malignant and 89 benign LNs in the test set). In this study, the fivefold crossover method was used to fit and verify the logistics model. The evaluation method included an ROC curve with AUC, ACC, SE, and SP. The software used for radiomics processing and model building was Python 3.6 (https://www.python.org/).

### Subjective diagnosis

Three radiologists with different rectal cancer diagnosis experience (senior: 6 years, Dr. Li; middle-aged: 3 years, Dr. Song; junior: 0.5 years, Dr. Jia) interpreted each LN on HR-T2WI independently without knowing the final pathological results. Taking the pathological results as the gold standard, the diagnostic efficacy of each radiologist was analyzed and compared. The method to evaluate the diagnostic efficacy was through an ROC curve with indexes including AUC, ACC, SE, and SP.

### Statistical methods and software

SPSS 20 (IBM Corp., Armonk, NY) and Medcalc 15.6.1 (Medcalc software Ltd.) software were used for statistical analysis. The measurement data conforming to normal distribution were expressed as mean ± standard deviation (SX). (1) Intra-class correlation coefficient (ICC) was used to evaluate the consistency of ROI segmentation in radiomics. ICC < 0.4: poor consistency; 0.4 < ICC < 0.75: the consistency is generally good; ICC > 0.75: very good consistency (23). (2) According to the results of homogeneity of variance, *t*-test or *T*’ test was used to analyze the difference between the short diameters of benign and malignant LNs, and the Youden index was used to determine the optimal threshold. (3) The DeLong test was used to compare the difference of AUCs between four kinds of ROI segmentation methods or between three radiologists’ subjective diagnosis. The evaluation method of radiomics and subjective diagnosis was ROC curve with indexes including AUC, ACC, SE, and SP. *p* < 0.05 was considered the threshold of statistical difference.

## Results

### Patient and LN cohort

A total of 449 patients with rectal neoplastic diseases were collected in this study. After screening by exclusion criterion 1, 102 cases of non-rectal cancer and 10 cases who could not accept MRI examination were excluded. Therefore, a total of 337 people underwent MR examination in this study. Among these 337 people, 120 cases were excluded after screening by exclusion standard 2, including 15 cases with poor image quality, 59 cases without visible LNs in the mesentery, and 46 cases without TME surgery for various reasons. Finally, 217 patients with visible LNs in the mesorectum were obtained and planned to undergo TME surgery. For patients with postoperative pathological results of N0, we believe that all visible LNs in mesorectal fascia (MRF) in preoperative MR images are negative.

**Figure 4:**
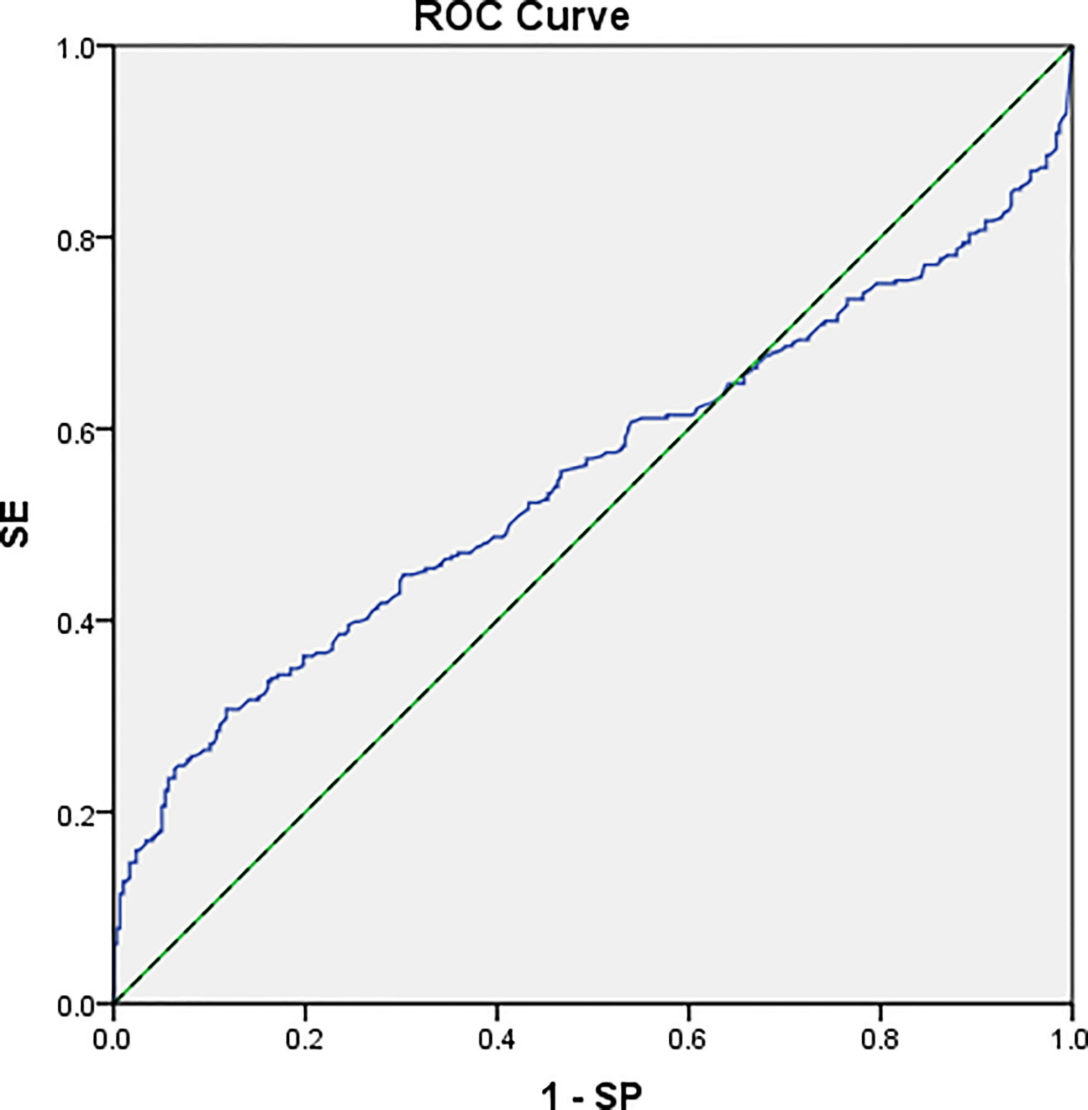
The ROC curve of using a short diameter as the diagnostic index. AUC = 0.552 (95% CI: 0.503–0.597).

Of these 217 cases, 51 patients had no correspondence between postoperative specimens and preoperative MR LNs. Finally, 166 patients were included in this study, and a total of 604 LNs were obtained, including 306 malignant and 298 benign lesions. The specific process is shown in **Supplementary Figure 2**. The age, sex, T and N stages, and the tumor position of the 166 patients are shown in **Table 2**. All tumors were pathologically confirmed as rectal adenocarcinoma after operation. Most of the short diameters of LNs were below 6 mm, accounting for 79.14%. The short diameter of malignant LNs was larger than that of benign LNs, and the difference was statistically significant (*p* < 0.05). However, when the short diameter was used as the diagnostic criterion, the AUC was 0.552. When the threshold was 5.93, the sensitivity was 0.307 and the specificity was 0.883 (**Table 3**; **Figure 4**).

**Table 2:** Clinicopathological parameters of 166 patients.

Clinicopathological parameters	Cases
Gender	
Male	102
Female	64
Age	
≤30	1
30–40 (40)	5
40–50 (50)	18
50–60 (60)	37
60–70 (70)	69
70–80 (80)	31
>80	5
T stage	
T1	4
T2	35
T3	104
T4	23
N stage	
N0	69
N1	51
N2	46
N+	97
Tumor position	
High	33
Medium	81
Low	52
Total	166

**Table 3:** Comparison of malignant and benign LNs.

Status	Number	Short diameters (mm)	M ± SD (mm)	*p*	AUC	SE	SP
Benign	298	3–9.42	4.83 ± 1.09	0.000	0.552	0.307	0.883
Malignant	306	3–14	5.45 ± 2.17				

### Intra- and inter-group consistency test

It can be found that the number of features whose ICC values were less than 0.75 within groups was lower than that between groups in all four methods (86 vs. 108 in method 1, 145 vs. 155 in method 2, 265 vs. 347 in method 3, and 232 vs. 333 in method 4). In segmentation method 1, the number of features with ICC value less than 0.75 was the least, both within and between groups. The feature values with ICC greater than 0.75 were included in the study, which can ensure the good consistency of feature extraction within and between observers.

### Data dimensionality reduction

**Figure 5:**
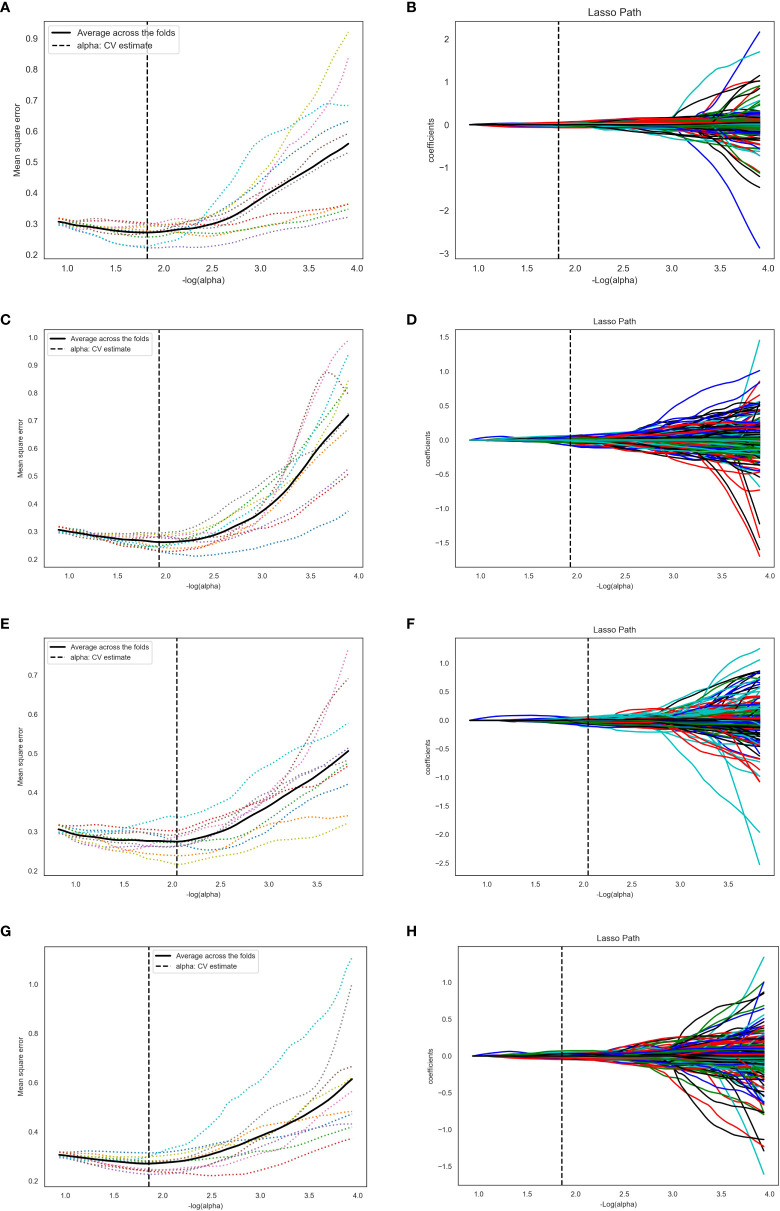
The process of Lasso dimension. **(A, B)**, **(C, D)**, **(E, F)**, and **(G, H)** represent ROI segmentation methods 1, 2, 3, and 4, respectively. For method 1, log(alpha) was 1.827 and alpha was 0.0149. For method 2, log(alpha) was 1.931 and alpha was 0.0117. For method 3, log(alpha) was 2.047 and alpha was 0.0089. For method 4, log(alpha) was 1.857 and alpha was 0.0139.

The final residual features of the four ROI segmentation methods were 45, 62, 65, and 35, respectively. **Figure 5** shows the process of Lasso dimension. The specific features of the four ROI segmentation methods after dimensionality reduction are shown in the attached Supplementary **Tables 2**–**5**.

### Logistic model fitting

The reduced dimension features were fitted to the logistic model. In this study, all 604 LNs were divided into a training set (207 benign and 215 malignant) and a test set (89 benign and 93 malignant) according to the ratio of 7:3.

**Figure 6:**
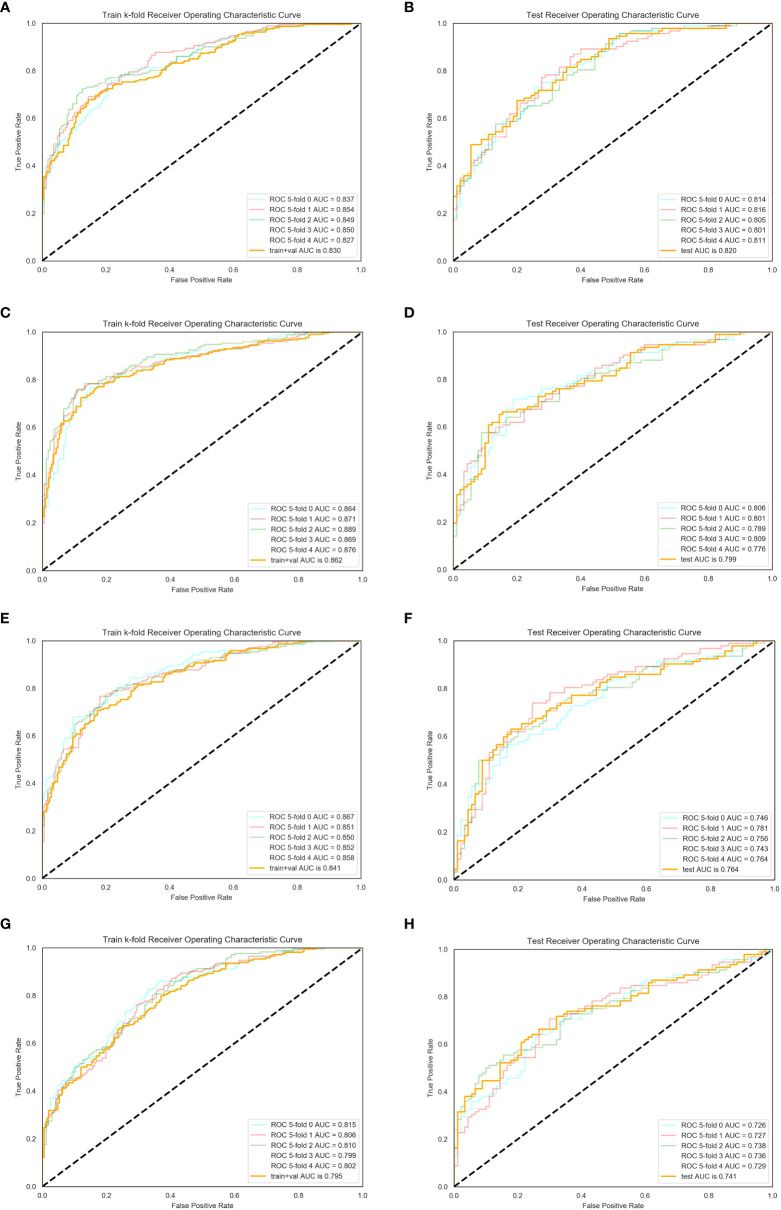
ROC of training and test sets. Fivefold cross-validation was used to assess the diagnostic efficiency. **(A, B)** For method 1, the mean AUCs of the training and test set were 0.830 and 0.820, respectively. **(C, D)** For method 2, the mean AUCs of the training and test set were 0.862 and 0.7999, respectively. **(E, F)** For method 3, the mean AUCs of the training and test set were 0.841 and 0.764, respectively. **(G, H)** For method 4, the mean AUCs of the training and test set were 0.795 and 0.741, respectively.

The features obtained by the four ROI segmentation methods were respectively fitted to the logistics model. Fivefold cross-validation with the index of AUC, accuracy, sensitivity, and specificity was used to evaluate the diagnosis efficiency of the models, which is shown in **Table 4**. In all test sets, the values of AUC, accuracy, and sensitivity obtained by segmentation method 1 were the highest (0.82, 0.725, and 0.756), and the value of specificity obtained by segmentation method 2 was the highest (0.772). **Figure 6** shows the ROC curves of the training set and verification set corresponding to the four ROI segmentation methods. There was no statistically significant difference in AUC between the four methods (*p*method 1 vs method 2 = 0.624, *p*method 1 vs method 3 = 0.2461, *p*method 1 vs method 4 = 0.1053, *p*method 2 vs method 3 = 0.4723, *p*method 2 vs method 4 = 0.2361, *p*method 3 vs method 4 = 0.6374).

**Table 4:** ROC analysis by LR of the training and test sets.

ROI segmentation	group	AUC	ACC	SE	SP
Method 1	Training set	0.830	0.851	0.819	0.824
	Test set	0.820	0.725	0.756	0.696
Method 2	Training set	0.862	0.882	0.904	0.764
	Test set	0.799	0.704	0.634	0.772
Method 3	Training set	0.841	0.851	0.821	0.880
	Test set	0.764	0.709	0.700	0.717
Method 4	Training set	0.795	0.753	0.763	0.794
	Test set	0.741	0.670	0.589	0.750

### Subjective diagnosis

**Figure 7:**
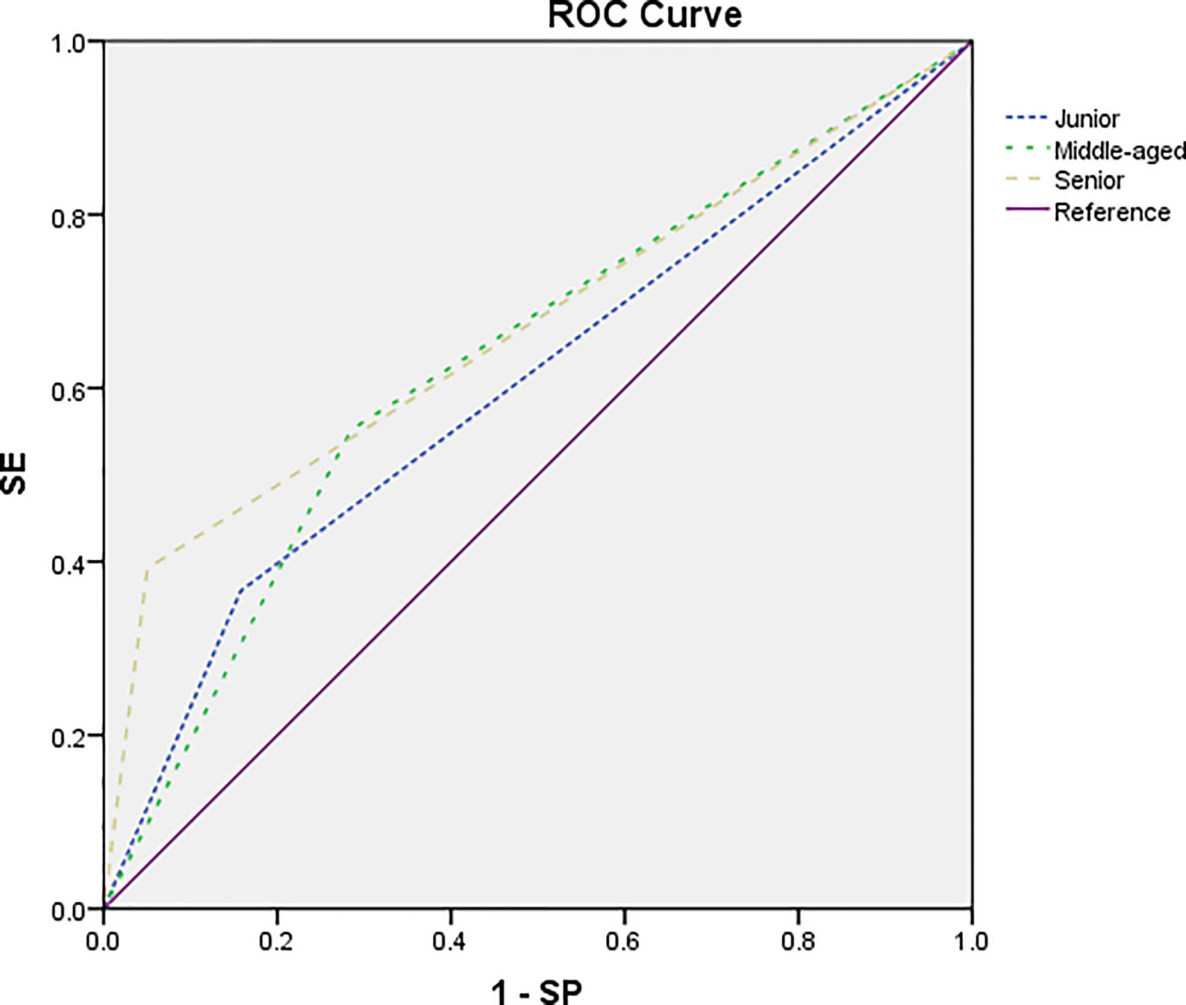
The ROC curve of the subjective diagnosis on nodes by a junior radiologist (short dotted line), a middle-aged radiologist (midpoint dotted line), and a senior radiologist (long dotted line). The AUC was estimated to be 0.604, 0.634, and 0.671, respectively.

Among the three radiologists, the AUC value (0.671) and ACC value (0.667) of the senior radiologist were the highest, followed by the middle-aged and junior ones. The diagnostic sensitivity of subjective diagnosis of the three radiologists was low, while the specificity was high. The diagnostic difference among the senior, junior, and middle-aged radiologist was statistically significant (senior vs. junior, *p* = 0.000; senior vs. middle-aged, *p* = 0.047), while the difference between the junior and middle-aged radiologist was not statistically significant (*p* = 0.181). See **Table 5** and **Figure 7** for the specific results.

**Table 5:** The subjective results of three radiologists.

Experience	Diagnosis	Pathological result		Total604	AUC	ACC	SE	SP
		Malignant 306	Benign 298				
Junior	Malignant	112	47	159	0.604	0.601	0.366	0.842
	Benign	194	251	445		
Middle-age	Malignant	169	85	254	0.634	0.632	0.552	0.715
	Benign	137	213	350		
Senior	Malignant	120	15	135	0.671	0.667	0.392	0.950
	Benign	186	283	469		

## Discussion

This study used radiomic signature to analyze the LNs within the mesorectum on HR-T2WI of rectal cancers. We collected 604 LNs from 166 patients with rectal cancer. Four kinds of segmentation methods were used to draw the ROI and the effect on the results was compared. By establishing the prediction model, we found that the efficiency of radiomic signature in the diagnosis of benign and malignant mesorectal LNs was higher than that of manual diagnosis. The method of ROI segmentation along the edge of LNs improved the stability of features and increased the diagnostic efficiency of the prediction model.

### Size criterion for LN involvement in preoperative MRI staging

At present, the size of LNs is one of the main features for MRI to judge LN metastasis. Malignant LNs increase in volume due to the invasion and appreciation of tumor cells. However, benign and malignant LNs overlap in size. Large LNs may be caused by reactive hyperplasia, with no invasion of cancer cells, while small LNs may be combined with micro-metastasis, which have no change in morphology and volume (33). This overlap leads to a failure agreement on the threshold of size of benign and malignant LNs (19, 20). In this study, the range in short diameter of 298 benign LNs in the mesorectum was 3–9.42 mm, with an average of 4.83 ± 1.09 mm, and that of 306 malignant LNs was 3–14 mm, with an average of 5.45 ± 2.17 mm. A significantly shorter average diameter was found in benign than in malignant LNs. When the threshold was set as 5.52 mm, the AUC, sensitivity, and specificity were 0.55, 0.307, and 0.883, respectively, showing insufficient diagnostic efficacy as a single index. This was similar to the research of Brown et al. (21), in which the threshold was set as 5 mm, the sensitivity was 42%, and the specificity was 87%. Therefore, when the size of LN is used as a single diagnostic standard, the diagnostic efficiency may not meet the clinical needs.

### The image selection and ROI segmentation for radiomic signature

Radiomics is one of the research hotspots in recent years. One first essential step in radiomics analysis is to determine suitable input imaging data. The main MRI techniques applied for rectum in the clinic include HR-T2WI, T1WI with and without image contrast administration, and DWI. One well-acknowledged method is the HR-T2w fast spin echo imaging, which can clearly display the edge, contour, and internal signals of LNs, and used as an important basis for the diagnosis of benign and malignant LNs (18, 21, 34, 35). Large-field T1w fast spin echo sequence (section thickness, 5 mm; field of view, 32 cm) is also important for finding LNs or related pelvic abnormalities, but it is difficult to characterize the lesions. Diffusion-weighted imaging (DWI) (*b* value = 500–1,000 s/mm^2^, section thickness = 5 mm) including apparent diffusion coefficient (ADC) map has also been included in our routine scheme as an auxiliary means of T2-weighted imaging to help evaluate post-treatment response or recurrence. However, the DWI sequence has low resolution, which cannot display the characteristics of LN edge and internal signal well, and cannot accurately distinguish benign reactive hyperplasia and LN metastasis (36). With gadolinium administration, contrast-enhanced T1WI, however, has not been shown to improve the accuracy of T- and N-stage tumor detection (37–39). Therefore, we believe that HR-T2WI is the most suitable sequence for radiomic signature.

Secondly, ROI segmentation is one of the most critical steps, because many extracted features depend on the segmented region. Whether the information with diagnostic value is included in ROI and whether the interference information without diagnostic value is excluded by ROI affect the diagnostic results of the radiomics model (30). Although the manual segmentation of ROI by radiologists can easily cause high differences between observers and the process is also time-consuming, it is still considered to be the “gold standard” (21, 35). Therefore, choosing an appropriate ROI segmentation method with certain repeatability will have a good impact on the results.

**Figure 8:**
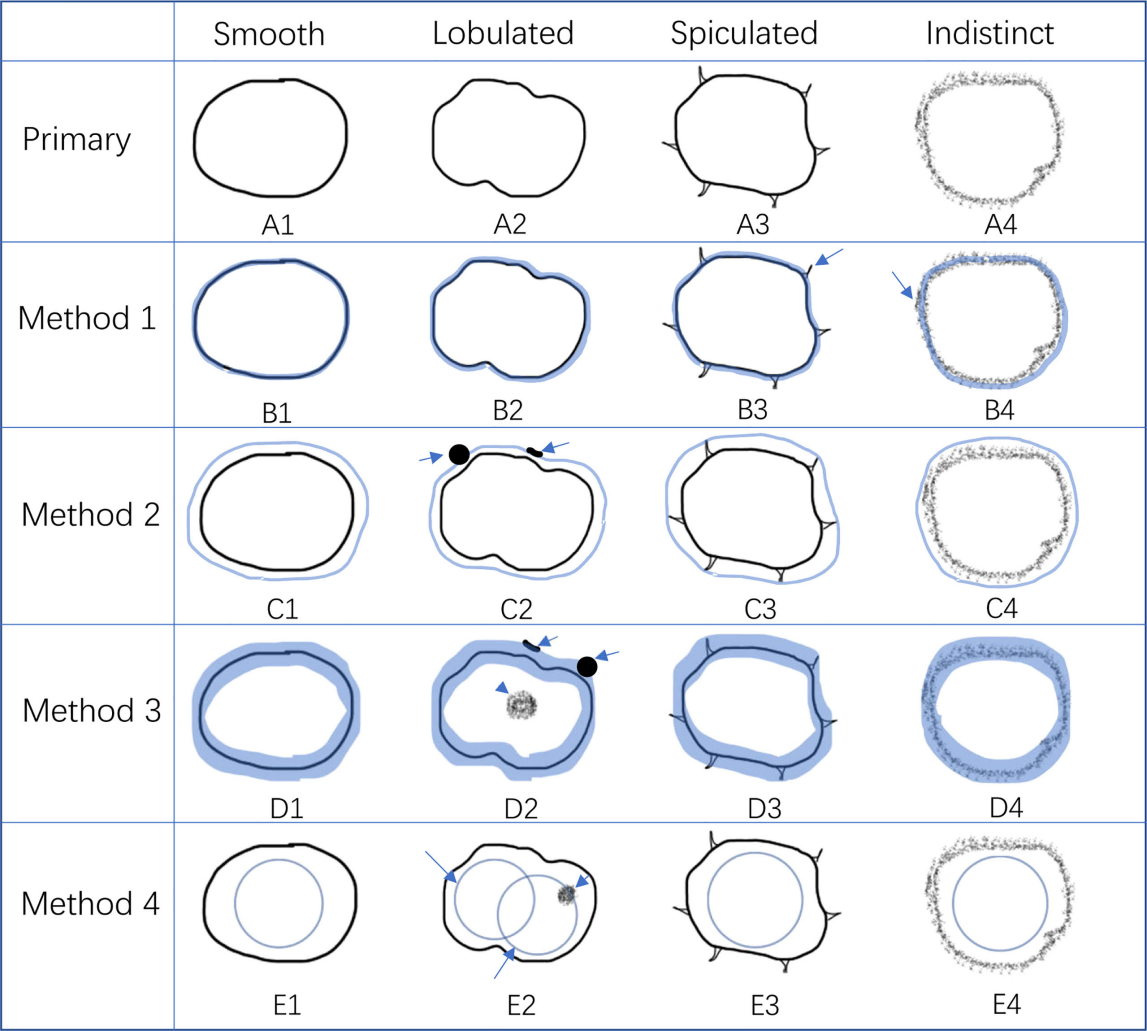
The advantage and disadvantage of four kinds of ROI segmentation methods. **(A1–4)** represent the characteristics of different borders of LNs, including smooth, lobulated, spiculated, and indistinct, according to Kim et al. (23). **(B1–4)** represent method 1 of ROI segmentation. It had good performance in smooth and lobulated border **(B1, 2)** but the burr of the spiculated border and part of the indistinct edge of the indistinct border were out of the ROI (**B3**, **4**, long arrow). **(C1–4)** represent method 2 of ROI segmentation. Although it covers more information than method 1 **(C3, 4)**, it may not reflect the border of the LNs as well as method 1. It may contain some non-diagnostic information, such as adjacent small blood vessels and other LNs (**C2**, long arrow). **(D1–4)** represent method 3 of ROI segmentation. It only contains the characteristic of the border without the internal signal (**D2**, short arrow). It may also cover some non-diagnostic information (**D2**, long arrow). **(E1–4)** represent method 4 of ROI segmentation. It only contains part of the internal signal of LNs without the border characteristic. The position of the ROI may affect the inclusion of the diagnostic part between the LNs (**E2**, long and short arrows).

As mentioned above, for the involved LNs, tumor infiltration within the LNs can lead to local exotropism, resulting in lobular changes (21). The reactive connective tissue hyperplasia generated after the tumor infiltrates the LNs or the tumor directly infiltrates the adipose tissue around the LNs, resulting in spiculated or indistinct changes (18). The high signal is caused by tumor tissue infiltration and necrosis or extracellular mucin mixed with the isosignal of residual normal LN tissue, resulting in internal signal nonuniformity (21). These pathological changes proved that the border contour and internal signal of LNs can be used to judge the status of LNs. Border contour can be subdivided into smooth or irregular. The irregular characteristic can be further subdivided into the lobulated, spiculated, and indistinct type (18) (**Figures 8A1–4**). In terms of diagnostic efficiency, border contour > internal signal (18, 21). For further subdivision, lobulation ≈> internal signal >> spiculated ≈ indistinct (18, 21). In this study, we used four ROI segmentation methods. Method 1 was to segment ROI along the border of LNs, which includes the internal signals and lobulated features of LNs. However, when the border of the LNs was spiculated or indistinct, this segmentation method may not accurately cover the burr or fuzziness, and some information may be lost (**Figures 8B1–4**). Therefore, we increased the ROI range to obtain method 2, which expanded 2–3 mm along the visible edge of the LNs (**Figures 8C1–4**). In addition, methods 3 and 4 were used to separate the border contour of LNs from internal signal (**Figures 8D1–4, E1–4**).

By fitting the logistic model, although there was no statistically significant difference in AUC between the four methods, method 1 had the highest value of AUC, ACC, and SE, followed by method 2, method 3, and method 4. According to the previous assumptions, method 2 contains more border contour features and the diagnostic efficiency should be better than method 1 in theory. However, the values of AUC, ACC, and SE of the actual results were lower than those of method 1 (0.820 vs. 0.799, 0.725 vs. 0.704, and 0.756 vs. 0.634), and only the specificity was higher than that of method 1 (0.772 vs. 0.696). The study of Jörn Gröne et al. (18) showed that spiculated/indistinct borders were rarely seen in the LN-positive group. The combination of internal signal and spiculated/indistinct border did not significantly improve the diagnostic efficiency. Only the diagnostic specificity was improved, while the sensitivity was reduced. This may be the main reason why the specificity of method 2 in this study was improved, while other parameters are not as high as method 1. At the same time, ROI expansion inevitably contains some non-diagnostic information, such as adjacent small blood vessels, other LNs, or intestinal wall, which may also reduce the diagnostic efficiency (**Figures 8, C2**).

Method 3 only contains border contour information (**Figures 8, C1–4**). It loses the signal features inside LNs and may contain useless structures outside LNs (**Figures 8, D2**). Method 4 only contains part internal signals of LNs while losing the information of border contour (**Figures 8, E1–4**). At the same time, the position of circle ROI may not contain the specific part of diagnostic information (**Figures 8, E2**). At the same time, we found that the result of method 3 was better than that of method 4, which indicates that the diagnostic information contained in the border contour of LNs is higher than the internal signal, which is consistent with the research results of Brown et al. (21). Therefore, it can be explained that the diagnostic efficiency of methods 3 and 4 was lower than that of methods 1 and 2 and the efficiency of method 4 was lower than that of method 3. We believe that the combined diagnosis efficiency of internal signal and border contour is higher than that of any single criteria, which is also consistent with the research results of Brown et al. (21).

By analyzing the feature values after dimension reduction by the four methods, we found that those that can reflect the signal changes, such as texture features and first-order features, accounted for the majority of the remaining feature values. This may be because among the 1,409 feature values included in the study, the number of original texture features and first-order feature values was 93, and the number of high-order features after various transformations was 1,302. They accounted for 99% of the total features, which may be one of the reasons why the number of the two kinds of features included in the model was also large. Therefore, we believe that the use of signal changes, not only within the LNs, but also along the border of the LNs, to reflect the difference between benign and malignant LNs may account for a large proportion in this study. Secondly, in method 1 to method 3, there was only one shape feature finally fitted in the model, which was elongation. It reflects the divergence or extension of ROI, which can be understood as the long-to-short ratio of ROI. Kim et al. (40) showed that the long-to-short ratio of benign LNs was greater than that of malignant LNs, and the difference was statistically significant. However, in method 4, there was no elongation in the eigenvalues after dimension reduction. This was because the ROIs of method 4 were all round and there was no difference between the long and short diameters.

Through between and within ICC comparison, we found that the number of features with ICC value less than 0.75 was the least in method 1, which indicates that method 1 was more repeatable than other methods. We speculate that this was because in method 1, the contour of LNs provides a reference for segmentation. For different readers, the outline of the contour was stationary, which may make the segmentation process more repeatable. For methods 2, 3 and 4, there were more subjective factors in the segmentation process. In methods 2 and 3, the expanded distance and the width of ROI segmentation line were subjective. At the same time, attention should be paid to avoid irrelevant tissue structures outside LNs. In method 4, the placement position and the size of circular ROI may be varied by different readers. Although we found that the difference of the AUC between the four methods was not significant (*p* > 0.05), method 1 obtained the largest number of stability features and can be assisted by contour boundaries in the segmentation process. Therefore, we think that method 1 is superior to methods 2, 3, and 4.

### Radiomic signature and subjective diagnosis

In this study, three radiologists with different levels of working experience independently diagnosed the LNs. The results showed that the AUC, accuracy, and sensitivity were not high (AUC: 0.604, 0.634, and 0.671; ACC: 0.601, 0.632, and 0.667; SE: 0.366, 0.552, and 0.392), while the specificity was high (0.842, 0.715, and 0.950). The diagnostic efficacy of the senior radiologist was higher than that of the low and middle-aged radiologists, and the difference was statistically significant (*p* < 0.05). The diagnostic efficacy of the middle-aged radiologist was higher than that of the junior radiologist, but the difference was not statistically significant (*p* > 0.05). This shows that it is difficult to diagnose the benign and malignant mesorectal LNs in clinical work. The overlap of LN size and the subjectivity of border contour and internal signals lead to the heterogeneity and unreliability of diagnosis between different radiologists (18, 41). In this study, the sensitivity of subjective diagnosis is low while the specificity is high, which is the result when the size of LNs was set as the only diagnostic criterion (SE = 0.307, SP = 0.883). We speculate that this is because in this study, the short diameter of most LNs is less than 5 mm. With the decrease of LN volume, its internal signal and border contour become challenging to be obtained by subjective assessment. Therefore, radiologists prefer to use the size of LNs as the diagnostic criterion, which leads to its diagnostic efficiency close to that of using LN short diameter as the criterion. In the clinic, lower sensitivity will lead to missed diagnosis of malignant LNs. Higher sensitivity is helpful to find suspicious LNs and reduce missed diagnosis, so as to improve the prognosis of patients after operation. Through the comparison between imaging radiomics and subjective diagnosis, we found that the overall evaluation index values of radiomics combined with logistics model were higher than those of subjective diagnosis. This shows that radiomic signature improves the diagnostic efficiency of malignant LNs.

### Compared with other studies

At present, there are relatively few studies on the application of radiomics in the diagnosis of benign and malignant LNs of rectal cancer. Li et al. (42) collected 132 LNs from 91 patients, including 86 malignant and 46 benign, with an average diameter of 8 and 9 mm, respectively. After fitting the model, they found that the accuracy, specificity, and sensitivity of the subjective diagnosis, radiomics model, and radiomics combined with subjective diagnosis were 72.09%, 73.81%, and 78.12%; 89.81%, 82.57% and 87.77%; and 92.23%, 84.69%, and 89.88%, respectively. The AUC of radiomics combined with subjective diagnosis was 0.94. The results of Li’s study are better than those of this study. We believe that this may be because the average size of benign and malignant LNs in Li’s study was between 8 and 9 mm, which is larger than that of LNs in our study (4–6 mm). Therefore, it has a better display of internal signals and border contour of LNs than this study. This may improve the accuracy of subjective diagnosis and ROI segmentation. Therefore, both the results of the subjective diagnosis and radiomics model are better than those of our study. In addition, by comparing the relevant studies of rectal cancer LNs, we found that, in most studies, the average size of LNs is less than 8 mm (18, 22, 23, 43), and the proportion of LNs above 8 mm is relatively low (18, 21). At the same time, malignant LNs account for the vast majority of LNs above 8 cm (41). This is consistent with this study; that is, LNs above 8 mm account for only 6.12% of the total, and malignant LNs above 8 mm account for 94.59%. Therefore, we believe that this study may be more representative in LNs with a short diameter of less than 8 mm. Although there are differences between Li’s study and this study, both studies have concluded that the ability of radiomic signature to identify benign and malignant LNs of rectal cancer is better than subjective diagnosis. In addition, this study did not combine radiomics with subjective diagnosis. In this study, it has been confirmed that subjective diagnosis varies from person to person. The model trained by subjective diagnosis results may only be applicable to radiologists who provide subjective diagnosis information. If the model was fitted to other radiologists, the prediction results of the model may become worse.

## Limitations

This study has the following shortcomings: The number of cases was small and there was a lack of multicenter research for verification; the structural changes and volume contraction of postoperative specimens will lead to the inaccuracy of localization, which may also be the problem faced by most of the current research on the diagnosis of LNs; LNs less than 3 mm were not included. Future research should address these limitations in order to achieve better results.

## Conclusion

Radiomic signature based on HR-T2WI is helpful in distinguishing metastatic LNs from non-metastatic LNs within the mesorectum of rectal cancer and has the potential ability to help doctors to make treatment decisions individually.

## Data availability statement

The raw data supporting the conclusions of this article will be made available by the authors, without undue reservation.

## Ethics statement

The studies involving human participants were reviewed and approved by the ethics committee of Qianfo Mountain hospital in Shandong Province. The patients/participants provided their written informed consent to participate in this study. Written informed consent was obtained from the individual(s) for the publication of any potentially identifiable images or data included in this article.

## Author contributions

AL and GS conceived and designed the experiments; AL, GS, YJ, and YH performed the experiments; GS and RZ analyzed the data; PL, RW, and RH contributed with patient material; and GS and JL wrote the paper. All authors read and approved the final manuscript.

## Funding

This article received a grant (201907064) from Jinan Science and Technology Plan.

## Acknowledgments

The authors acknowledge the help of Huiying Medical Technology Co. Ltd.

## Conflict of interest

Author RZ was employed by Huiying Medical Technology Co., Ltd. Beijing, China.

The remaining authors declare that the research was conducted in the absence of any commercial or financial relationships that could be construed as a potential conflict of interest.

## Publisher’s note

All claims expressed in this article are solely those of the authors and do not necessarily represent those of their affiliated organizations, or those of the publisher, the editors and the reviewers. Any product that may be evaluated in this article, or claim that may be made by its manufacturer, is not guaranteed or endorsed by the publisher.
