# Supplementary material for: Development and validation of a high-resolution T2WI-based radiomic signature for the diagnosis of lymph node status within the mesorectum in rectal cancer
Source: Front Oncol. 2022 Sep 16;12:945559. doi: 10.3389/fonc.2022.945559 (PMC9523667; doi:10.3389/fonc.2022.945559)
Supplement: Supplementary file 1 [file DataSheet_1.docx]

Supplementary Material

# Supplementary Figures and Tables

Table 1 the features included in this study

| Associated filter | Radiomic group | Radiomic features |
| --- | --- | --- |
| Original  Logarithm  Exponential  Gradient  Square  Squareroot  Lbp-2D  Wavelet-LHL  Wavelet-LHH  Wavelet-HLL  Wavelet-LLH  Wavelet-HLH  Wavelet-HHH  Wavelet-HHL  Wavelet-LLL  (total 15) | First order(18) | Interquartile Range, Skewness, Uniformity, Median, Energy, RobustMeanAbsoluteDeviation, Mean Absolute Deviation, Total Energy, Maximum, Root Mean Squared, 90 Percentile, Minimum, Entropy, Range, Variance, 10Percentile, Kurtosis, Mean. |
|  | GLCM(24) | Joint Average, Sum Average, Joint Entropy, Cluster Shade, Maximum Probability, Idmn, Joint Energy, Contrast, Difference Entropy, Inverse Variance, Difference Variance, Idn, Idm, Correlation, Autocorrelation, Sum Entropy, MCC, Sum Squares, Cluster Prominence, Imc2, Imc1, Difference Average, Id, Cluster Tendency. |
|  | GLDM(14) | Gray Level Variance, High Gray Level Emphasis, Dependence Entropy, Dependence Non Uniformity, Gray Level Non Uniformity, Small Dependence Emphasis, Small Dependence High Gray Level Emphasis, Dependence NonUniformity Normalized, Large Dependence Emphasis, Large Dependence Low Gray Level Emphasis, Dependence Variance, Large Dependence High Gray Level Emphasis, Small Dependence Low Gray Level Emphasis, Low Gray Level Emphasis. |
|  | GLRLM(16) | Short Run Low Gray Level Emphasis, Gray Level Variance, Low Gray Level Run Emphasis, Gray Level Non Uniformity Normalized, Run Variance, Gray Level Non Uniformity, Long Run Emphasis, Short Run High Gray Level Emphasis, Run Length Non Uniformity, Short Run Emphasis, Long Run High Gray Level Emphasis, Run Percentage, Long Run Low GrayLevel Emphasis, Run Entropy, High Gray Level Run Emphasis, Run Length NonUniformity Normalized. |
|  | Glszm(16) | Gray Level Variance, Zone Variance, Gray Level Non Uniformity Normalized, Size Zone Non Uniformity Normalized, Size Zone Non Uniformity, Gray Level Non Uniformity, Large Area Emphasis, Small Area High Gray Level Emphasis, Zone Percentage, Large Area Low Gray Level Emphasis, Large Area High Gray Level Emphasis, High Gray Level Zone Emphasis, Small Area Emphasis, Low Gray Level Zone Emphasis, Zone Entropy, Small Area Low Gray Level Emphasis. |
|  | Ngtdm(5) | Coarseness, Complexity, Strength, Contrast, Busyness. |
| Original | Shape(14) | Voxel Volume, Maximum3DDiameter, MeshVolume, Major Axis Length, Sphericity, Least Axis Length, Elongation, Surface Volume Ratio, Maximum2DDiameterSlice, Flatness, Surface Area, Minor Axis Length, Maximum2DDiameterColumn, Maximum2DDiameterRow. |
| Total | | 15*(28+24+14+16+16+5)+14=1409 |

Table 2 the features after dimensionality reduction in method 1

| Associated filter | Radiomic group | Radiomic features |
| --- | --- | --- |
| gradient | firstorder | Kurtosis,InterquartileRange  90Percentile |
|  | gldm | DependenceVariance |
| original | gldm | DependenceNonUniformityNormalized |
|  | shape | Elongation |
|  | firstorder | 90Percentile |
|  | glrlm | LongRunHighGrayLevelEmphasis |
| exponential | gldm | DependenceVariance |
| square | gldm | DependenceVariance |
| logarithm | gldm | DependenceNonUniformityNormalized |
| squareroot | firstorder | RootMeanSquared |
| wavelet-LHL | ngtdm | Busyness |
|  | glszm | GrayLevelNonUniformity |
|  | firstorder | Skewness,Mean,Variance |
|  | glcm | Correlation |
| wavelet-HLL | glszm | SmallAreaHighGrayLevelEmphasis |
|  |  | GrayLevelNonUniformity |
|  | firstorder | Minimum,10Percentile |
|  | gldm | LargeDependenceLowGrayLevelEmphasis |
| wavelet-HHH | glszm | SizeZoneNonUniformity |
|  | ngtdm | Busyness |
| wavelet-LLL | firstorder | 10Percentile |
|  | ngtdm | Contrast |
|  | glszm | SmallAreaEmphasis  GrayLevelNonUniformity |
|  | glcm | ClusterTendency |
|  | gldm | DependenceEntropy |
| wavelet-LHH | glrlm | GrayLevelNonUniformityNormalized  ShortRunEmphasis |
|  | glszm | SizeZoneNonUniformityNormalized |
|  | firstorder | Median,Minimum |
|  | glcm | Idm |
| wavelet-LLH | glszm | LargeAreaLowGrayLevelEmphasis  SmallAreaEmphasis  SizeZoneNonUniformity  SmallAreaHighGrayLevelEmphasis |
| wavelet-HLH | firstorder | Median |
|  | ngtdm | Busyness |
| wavelet-HHL | firstorder | Median,10Percentile |

Table 3 the features after dimensionality reduction in method 2

| Associated filter | Radiomic group | Radiomic features |
| --- | --- | --- |
| original | glszm | ZoneEntropy, SizeZoneNonUniformityNormalized, GrayLevelNonUniformityNormalized |
|  | glrlm | ShortRunHighGrayLevelEmphasis  LowGrayLevelRunEmphasis |
|  | glcm | MCC |
|  | shape | Elongation |
| square | firstorder | RootMeanSquared |
| gradient | firstorder | Kurtosis,MeanAbsoluteDeviation |
| squareroot | glrlm | ShortRunHighGrayLevelEmphasis  LowGrayLevelRunEmphasis |
|  | glcm | MCC |
|  | glszm | SizeZoneNonUniformityNormalized |
|  | firstorder | TotalEnergy, Median |
| logarithm | glrlm | ShortRunHighGrayLevelEmphasis |
|  |  | LowGrayLevelRunEmp hasis |
|  | glcm | MCC |
|  | glszm | SizeZoneNonUniformityNormalized |
| exponential | firstorder | Kurtosis, Variance |
| wavelet-LLL | glcm | ClusterShade |
|  | firstorder | Minimum, Kurtosis |
|  | glszm | GrayLevelNonUniformity,SmallAreaEmphasis |
|  | glrlm | RunEntropy |
| wavelet-LLH | glrlm | RunLengthNonUniformity |
|  | glszm | SizeZoneNonUniformityNormalized |
|  | firstorder | Variance |
| wavelet-HLL | glszm | GrayLevelNonUniformity  SizeZoneNonUniformityNormalized |
|  | glcm | JointAverage, SumAverage |
|  | glrlm | ShortRunHighGrayLevelEmphasis |
|  | firstorder | Median, Skewness |
| wavelet-HHH | firstorder | TotalEnergy |
|  | glrlm | ShortRunLowGrayLevelEmphasis |
|  |  | RunPercentage |
|  | ngtdm | Coarseness |
| wavelet-HHL | glrlm | ShortRunLowGrayLevelEmphasis |
|  | glcm | ClusterShade |
|  |  | ClusterTendency |
| wavelet-LHH | glszm | ZoneVariance |
|  | firstorder | TotalEnergy |
|  | glcm | ClusterProminence |
|  | gldm | LargeDependenceLowGrayLevelEmphasis |
|  | ngtdm | Coarseness |
|  | glrlm | ShortRunEmphasis |
| wavelet-LHL | glszm | LowGrayLevelZoneEmphasis  GrayLevelVariance |
|  | glrlm | HighGrayLevelRunEmphasis |
|  | firstorder | RootMeanSquared, 90Percentile |
|  | gldm | DependenceEntropy |
|  | gldm | HighGrayLevelEmphasis |
| wavelet-HLH | glrlm | LowGrayLevelRunEmphasis, ShortRunHighGrayLevelEmphasis |
|  | firstorder | Skewness |
|  | glszm | LargeAreaLowGrayLevelEmphasis |

Table 4 the features after dimensionality reduction in method 3

| Associated filter | Radiomic group | Radiomic features |
| --- | --- | --- |
| exponential | glszm | LargeAreaEmphasis |
|  | glrlm | RunVariance |
|  | firstorder | RootMeanSquared |
| lbp-2D | firstorder | TotalEnergy, Kurtosis |
| logarithm | glszm | ZonePercentage,ZoneEntropy,  SizeZoneNonUniformityNormalized,  LargeAreaLowGrayLevelEmphasis |
| logarithm | firstorder | Skewness, RootMeanSquared, 90Percentile |
| original | shape | Elongation |
|  | glszm | ZonePercentage, ZoneEntropy  SizeZoneNonUniformityNormalized  LargeAreaLowGrayLevelEmphasis |
|  | glrlm | ShortRunHighGrayLevelEmphasis |
|  | gldm | DependenceNonUniformityNormalized |
|  | glcm | ClusterProminence |
| square | firstorder | Range, InterquartileRange |
| squareroot | glszm | ZonePercentage, ZoneEntropy  SizeZoneNonUniformityNormalized  LargeAreaLowGrayLevelEmphasis |
|  | firstorder | Range, 10Percentile |
| wavelet-HHH | ngtdm | Busyness |
|  | glszm | LargeAreaHighGrayLevelEmphasis |
|  | gldm | DependenceNonUniformity |
|  | firstorder | Skewness |
| wavelet-HLH | glszm | ZoneEntropy |
|  | glcm | InverseVariance,Idn,Idmn,Contrast, ClusterTendency, DifferenceAverage  , ClusterProminence |
|  | firstorder | Range,Minimum,90Percentil |
| wavelet-LHH | ngtdm | Busyness |
|  | glszm | ZoneEntropy,SmallAreaLowGrayLevelEmphasis,  SizeZoneNonUniformityNormalized,  LowGrayLevelZoneEmphasis,  HighGrayLevelZoneEmphasis |
|  | glcm | ClusterProminence |
| wavelet-HLL | glszm | SizeZoneNonUniformityNormalized |
| wavelet-LHL | gldm | DependenceNonUniformity |
| wavelet-LLH | glszm | GrayLevelNonUniformity |
|  | firstorder | Variance,Skewness, firstorderMaximum,  RobustMeanAbsoluteDeviation,90Percentil |
| wavelet-LLL | ngtdm | Contrast,Busyness |
|  | glszm | GrayLevelNonUniformit |
|  | gldm | LargeDependenceLowGrayLevelEmphasis  DependenceEntropy |
| wavelet-HHL | ngtdm | Busyness |
|  | glszm | ZoneVariance |

Table 5 the features after dimensionality reduction in method 4

| Associated filter | Radiomic group | Radiomic features |
| --- | --- | --- |
| gradient | firstorder | RobustMeanAbsoluteDeviation |
| original | firstorder | Skewness |
|  | shape | Maximum2DDiameterColumn |
| square | firstorder | InterquartileRange,Skewness |
| squareroot | firstorder | RootMeanSquared |
| wavelet-HHH | firstorder | Median,TotalEnergy |
|  | glcm | DifferenceEntropy, DifferenceVariance |
| wavelet-HHL | glcm | ClusterProminence |
| wavelet-HHL | glszm | ZoneVariance |
| wavelet-HLH | firstorder | 90Percentile |
| wavelet-HLH | glszm | GrayLevelNonUniformity, ZoneEntropy |
| wavelet-LHH | firstorder | TotalEnergy |
|  | glrlm | RunLengthNonUniformity |
| wavelet-HLL | glcm | MaximumProbability |
|  | gldm | DependenceNonUniformityNormalized |
|  | glszm | GrayLevelNonUniformityNormalized, GrayLevelVariance |
|  | ngtdm | Contrast |
| wavelet-LHL | firstorder | 90Percentile |
|  | glcm | Autocorrelation |
|  | gldm | DependenceNonUniformity |
| wavelet-LLH | firstorder | RootMeanSquared, Variance |
|  | glszm | GrayLevelNonUniformity |
| wavelet-LLL | firstorder | Minimum,RootMeanSquared |
|  | glcm | Imc1 |
|  | gldm | DependenceEntropy |
|  | glrlm | LongRunHighGrayLevelEmphasis |
|  | ngtdm | Busyness, Strength |

Supplementary figures and figure legends


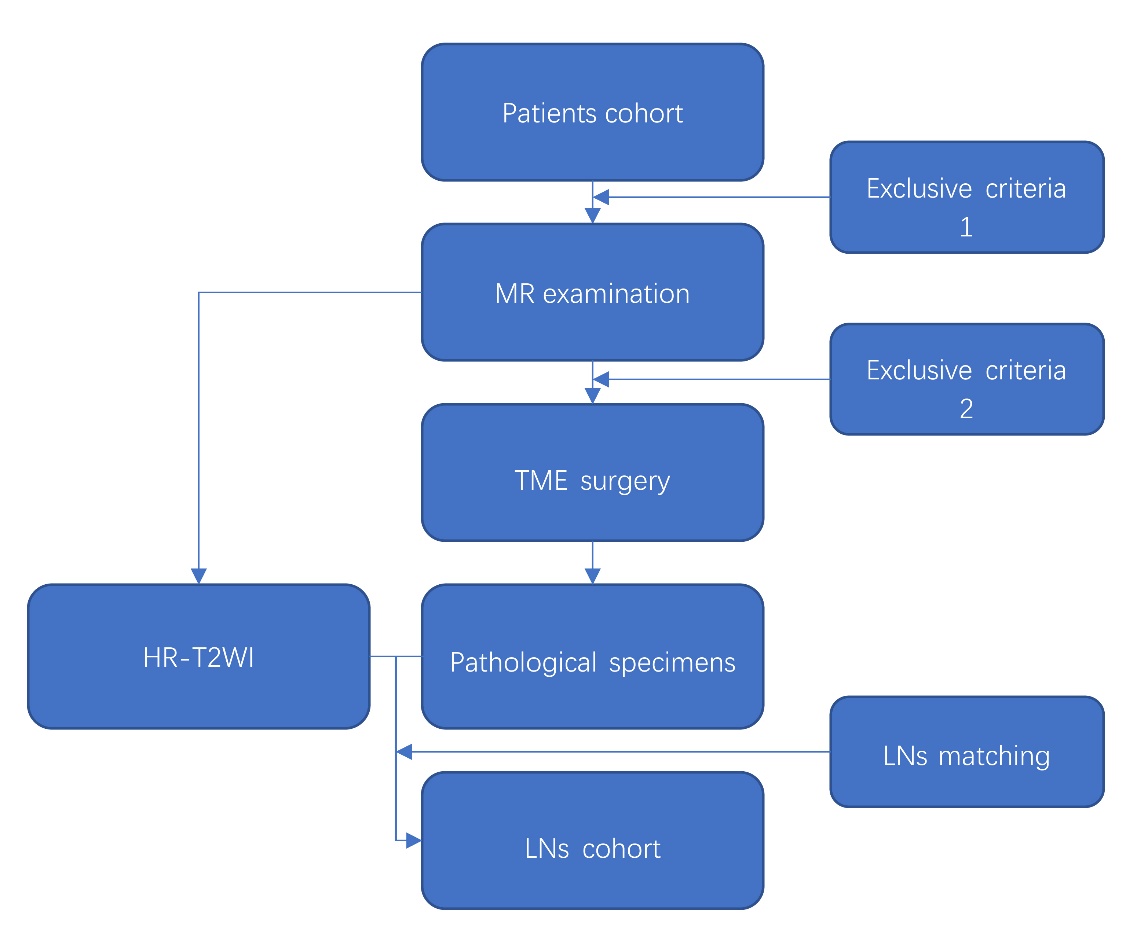


Supplementary figure 1 The establishment process of LN cohort


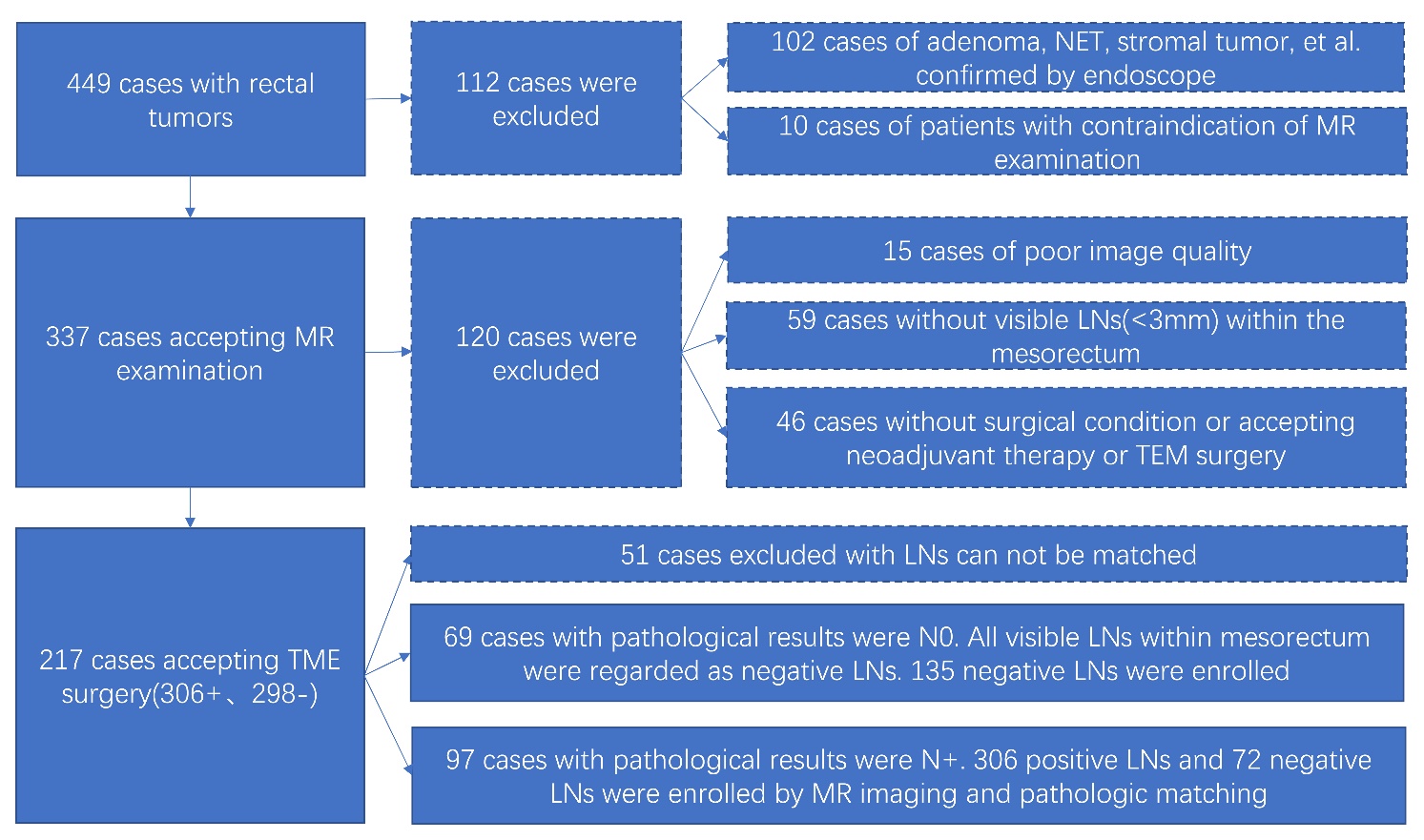
Supplementary figure 2 The specific process of the collection of LNs
